# Laser interstitial thermal therapy for the treatment of insular lesions: A systematic review

**DOI:** 10.3389/fneur.2022.1024075

**Published:** 2023-01-04

**Authors:** Artur Vetkas, Jürgen Germann, Alexandre Boutet, Nardin Samuel, Can Sarica, Kazuaki Yamamoto, Brendan Santyr, Cletus Cheyuo, Christopher R. Conner, Stefan M. Lang, Andres M. Lozano, George M. Ibrahim, Taufik Valiante, Paul N. Kongkham, Suneil K. Kalia

**Affiliations:** ^1^Division of Neurosurgery, Department of Surgery, University Health Network and University of Toronto, Toronto, ON, Canada; ^2^Neurology Clinic, Department of Neurosurgery, Tartu University Hospital, University of Tartu, Tartu, Estonia; ^3^Joint Department of Medical Imaging, University of Toronto, Toronto, ON, Canada; ^4^Schulich School of Medicine and Dentistry, Western University, London, ON, Canada; ^5^Krembil Research Institute, Toronto, ON, Canada; ^6^Division of Pediatric Neurosurgery, Sick Kids Toronto, University of Toronto, Toronto, ON, Canada; ^7^CRANIA, University Health Network and University of Toronto, Toronto, ON, Canada; ^8^The KITE Research Institute, University Health Network, Toronto, ON, Canada

**Keywords:** LITT, MRgLITT, insula, epilepsy, seizure, tumor, glioma

## Abstract

**Background:**

The surgical treatment of insular lesions has been historically associated with high morbidity. Laser interstitial thermal therapy (LITT) has been increasingly used in the treatment of insular lesions, commonly neoplastic or epileptogenic. Stereotaxis is used to guide laser probes to the insula where real-time magnetic resonance thermometry defines lesion creation. There is an absence of previously published reviews on insular LITT, despite a rapid uptake in use, making further study imperative.

**Methods:**

Here we present a systematic review of the PubMed and Scopus databases, examining the reported clinical indications, outcomes, and adverse effects of insular LITT.

**Results:**

A review of the literature revealed 10 retrospective studies reporting on 53 patients (43 pediatric and 10 adults) that were treated with insular LITT. 87% of cases were for the treatment of epilepsy, with 89% of patients achieving seizure outcomes of Engle I-III following treatment. The other 13% of cases reported on insular tumors and radiological improvement was seen in all cases following treatment. All but one study reported adverse events following LITT with a rate of 37%. The most common adverse events were transient hemiparesis (29%) and transient aphasia (6%). One patient experienced an intracerebral hemorrhage, which required a decompressive hemicraniectomy, with subsequent full recovery.

**Conclusion:**

This systematic review highlights the suitability of LITT for the treatment of both insular seizure foci and insular tumors. Despite the growing use of this technique, prospective studies remain absent in the literature. Future work should directly evaluate the efficacy of LITT with randomized and controlled trials.

## Introduction

Laser interstitial thermal therapy (LITT) is a thermoablative procedure that uses stereotactically implanted laser catheters to create a lesion that is precisely defined by the delivered energy using real-time magnetic resonance thermometry (1, 2). In recent years LITT has been adopted for the treatment of various insular lesions, including tumors and epileptic foci (3, 4). The surgical treatment of insular lesions is associated with high morbidity due to limitations imposed by the nearby opercular cortex and complex surrounding vascular anatomy (5, 6). Anatomically, the insula is adjacent to eloquent structures, such as the internal capsule and opercular cortex, and is encased by the branches of the middle cerebral artery (7, 8). Despite its name, translated as ‘island' from Latin, the insula is a highly connected brain region with projections to the orbitofrontal, temporal, parietal, supplementary motor areas, anterior cingulate, and subcortical regions (thalamus, globus pallidus, and amygdala) (9, 10). Insular lesions include gliomas, metastatic brain tumors, cavernous angiomas, and cortical dysplasia among others.

The treatment of deep seated brain tumors is often limited to chemo- and radiotherapy due to the high risk of permanent neurological deficit (11, 12). Since the most consistent prognostic factor for survival in gliomas is gross total resection, alternatives that can provide successful cytoreduction with limited invasiveness and surgical footprint are needed for treatment of deep seated brain tumors (13–15). Due to its less invasive nature, LITT may reduce vascular complications and spare the eloquent cortex while achieving cytoreduction or disconnection of epileptic circuits. Laser interstitial thermal therapy has been successfully used to treat insular tumors, multifocal lesions, and radiation necrosis (16–22).

Recently, through a wider use of stereotactic electroencephalography (SEEG), insular epilepsy has been recognized as a separate diagnostic entity (23). Insular LITT was used for the treatment of epileptic lesions both in pediatric and adult cohorts (24–26). Laser interstitial thermal therapy compares favorably with other minimally invasive therapies for the treatment of drug resistant epilepsy (DRE), such as radiofrequency thermocoagulation (RFTC) with SEEG electrodes and gamma knife surgery (GKS) (23, 26).

Laser interstitial thermal therapy is a novel and useful therapy for the treatment of deep-seated brain lesions and epilepsy. We sought to review the published literature on the topic of insular LITT and report on the clinical indications, outcomes, and adverse effects. To our knowledge, this is the first systematic review published on the use of LITT for treating insular lesions.

## Methods

**Figure 1:**
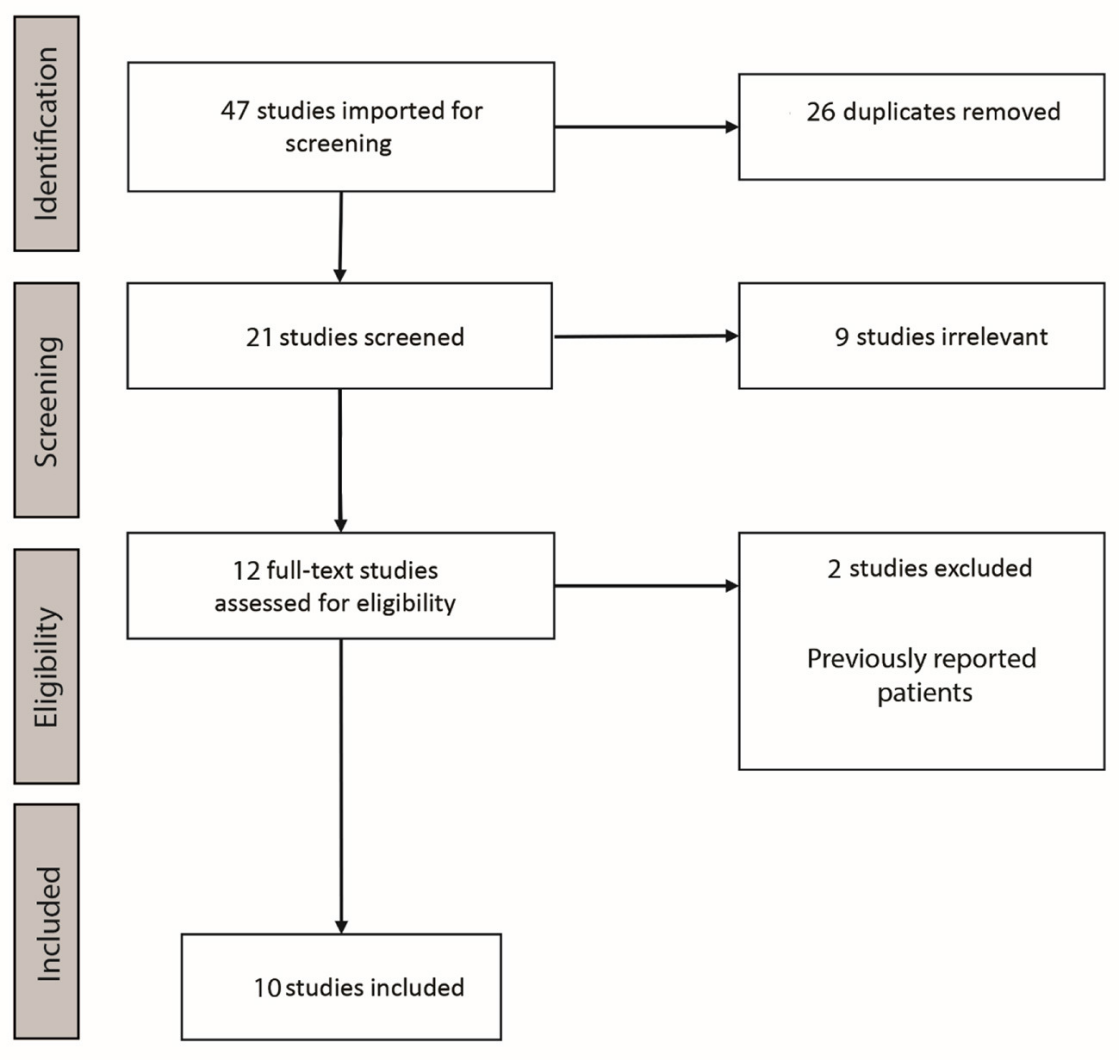
Flowchart of the systematic review for insular LITT.

We performed a systematic literature review on PubMed and Scopus databases using the following terms: “insula litt” (15), “insula laser interstitial” (17), “insular laser interstitial” (15), “MRgLITT insula” (2). The identified articles were screened by two independent authors (AV and KY) separately according to the PRISMA guidelines in April 2022. We included original studies published in English language. A total of 21 articles have been screened and 12 articles were identified matching the topic of this review. Two articles have been removed due to patients that have been previously reported (22, 27). Ten articles have been included in this review published from 2013 to 2022 (3, 22, 25–32). Pediatric patients were defined as those 18 years old or younger. Patient characteristics and available treatment parameters have been extracted from the included studies (Supplementary Table 1). The flowchart of the systematic review is presented in Figure 1.

## Results

A total of 10 studies that met inclusion criteria were identified. Seven studies reported outcomes of LITT in children and four in adults (one study included both adult and pediatric cases). In total, 53 patients have been reported as treated with insular LITT (43 children and 10 adults). 36% of the patients were female. The mean age was 12.5 (range 1.3–18) in the pediatric and 45.3 (range 53–73) years old in the adult group. Laser interstitial thermal therapy was used for the treatment of epilepsy in 87% and for insular tumor treatment in 13% of cases. The summary of the included studies and cohorts is presented in Table 1. The individual patient information and treatment characteristics are presented in Supplementary Table 1.

**Table 1:** Characteristics of the patients treated with insular LITT according to reported parameters.

	**Epilepsy**	**Tumor**
Studies	63.6% (*n =* 7)	36.4% (*n =* 4)
Cases	86.8% (*n =* 46)	13.2% (*n =* 7)
Pediatric	89.1% (*n =* 41)	28.6% (*n =* 2)
Adult	10.9% (*n =* 5)	71.4% (*n =* 5)
Previous surgery	60.9% (*n =* 28)	28.6% (*n =* 2)
Follow-up	18.9 mo	10.8 mo
**Outcome**		
Engel I	57% (*n =* 26)	–
Engel II	15% (*n =* 7)	–
Engel III	17% (*n =* 8)	–
Engel IV	11% (*n =* 5)	–
Trajectories	2.4 (*n =* 7)	2 (*n =* 2)
Mean ablation volume	8.4 cm3 (*n =* 10)	12.3 cm3 (*n =* 2)
Adverse effects	37.3% (19 out of 51)	
Transient paresis	5.8% (*n =* 3)	
Transient aphasia	29.4% (*n =* 15)	
Transient dysphagia	2% (*n =* 1)	

### Laser interstitial thermal therapy for insular epilepsy

**Figure 2:**
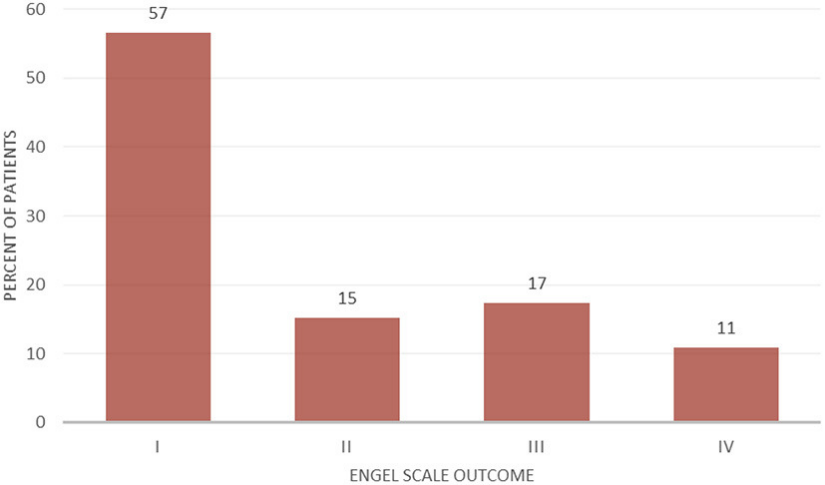
Engel scale seizure outcome after insular LITT for epilepsy (*n* = 46).

Laser interstitial thermal therapy was used for the treatment of insular epilepsy in 46 cases. 89.1% of the epilepsy LITT cases were pediatric and 10.9% were adults. Previous surgical treatment was reported in 60.9% of epilepsy patients. Mean follow-up for patients with epilepsy was 18.9 months. 57% of the patients had an Engel I outcome, while 89% of the patients had a seizure outcome of Engel I–III (Figure 2).

### Laser interstitial thermal therapy for insular tumors

Laser interstitial thermal therapy was used for the treatment of insular tumors in seven patients of whom 71.4% were adults. Previous resective treatment was reported in two of the tumor cases. Mean follow-up for patients with tumors was 10.8 months. Three of the patients presented with glioblastoma, two grade II gliomas, one colon adenocarcinoma metastasis and one atypical teratoid/rhabdoid tumor. Radiological improvement was reported for all cases. The patient with low grade glioma recurrence achieved seizure freedom after LITT.

### Technical parameters

Treatment parameters were presented for 12 patients with epilepsy. A mean of 2.4 trajectories were used (range 2–3). A mean ablation volume of 8.4 cm^3^ (range 1.02–29.2 cm^3^) was achieved. The ablation energy parameters ranged from 6 to 10.5 W for 90–180 s.

Treatment parameters were presented in three patients with insular tumors. Two trajectories were used and a mean ablation volume of 12.3 cm^3^ (9.9 and 14.6 cm^3^) was achieved in two patients. The ablation energy parameters were presented for one case (78 W, 768 s total).

**Figure 3:**
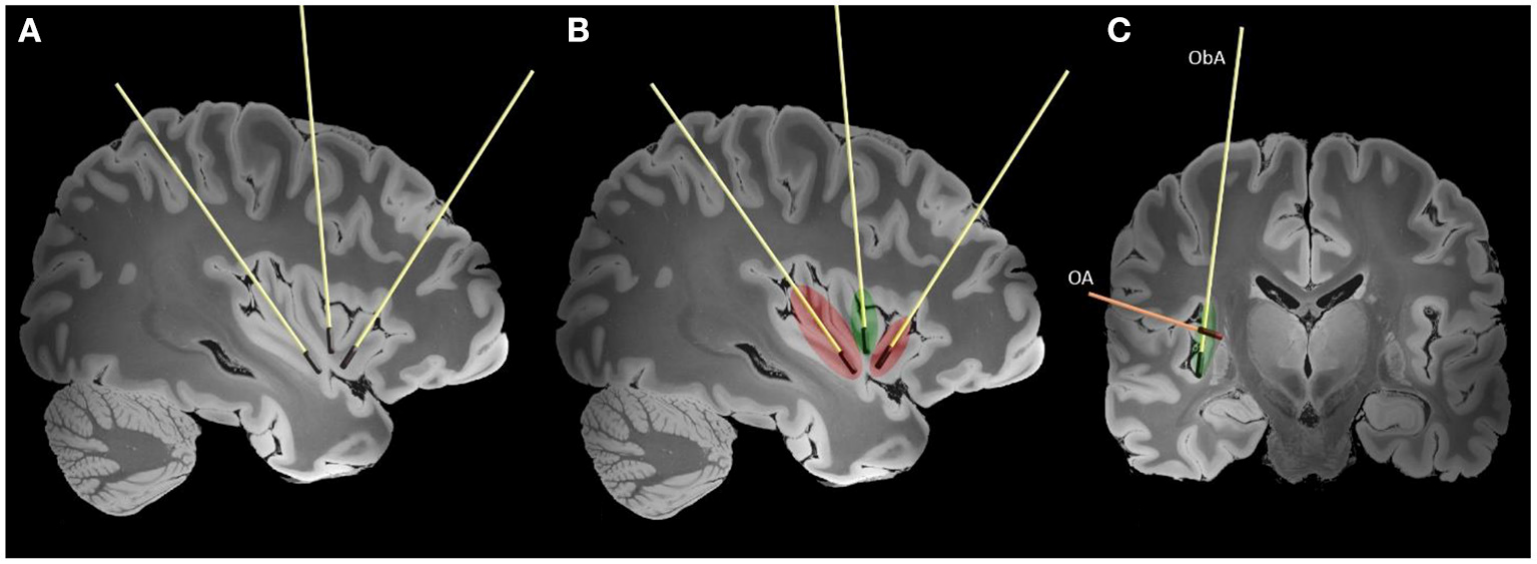
Typically used trajectories for the LITT of the insula are shown on high-resolution 7-tesla T1 magnet resonance imaging (MRI) slices of the human brain (38). Laser interstitial therapy trajectories are shown on sagittal **(A, B)** and coronal **(C)** slices. The red color represents lesions created by the anterior and posterior laser probes and extent of the disconnection. The green color represents the additional ablation needed to achieve complete insular ablation. In figure the two main LITT trajectories are presented (ObA—oblique parasagittal approach, OA—orthogonal trans-opercular approach).

As shown in Figure 3, the main trajectories that have been used for insular LITT were the orthogonal (axially oriented trans-opercular catheters) and parasagittal oblique (anterior and posterior).

### Adverse effects of insular laser interstitial thermal therapy

Adverse effects of LITT were reported in 10 studies and occurred in 37% (19 out of 51) of cases. Transient aphasia was present in 6% (3 out of 50) and transient hemiparesis in 29% (15 out of 51). One patient experienced dysphagia that resolved by 3-months. One pediatric patient had a large intracranial hemorrhage that required a decompressive craniectomy with subsequent bone flap replacement and recovery of paresis at 1 year follow-up.

## Discussion

We performed a systematic review of LITT therapy for lesions located within the insula. Our results indicate that seizure reduction after LITT is similar to conventional open surgical techniques. Laser interstitial thermal therapy is a novel minimally invasive alternative for the treatment of deep seated brain tumors, including those located in the insula. The two main indications for insular LITT are tumors and lesional epilepsy. Laser interstitial therapy is associated with minimal postoperative stay and a shorter hospital stay.

### Laser interstitial thermal therapy for insular epilepsy

Seizure onset within the insula is increasingly recognized as a cause of intractable epilepsy (23). There are approximately 150 insular epilepsy cases described in the literature based on SEEG studies (23). The EEG evaluation of insula is challenging due to its location and overlying cortex. The recent recognition of insular epilepsy as a new entity is associated with the implementation of invasive diagnostics. It is plausible that undiagnosed insular epilepsy may have been associated with previously failed epilepsy resections (23, 28). The semiology of insular seizures is heterogenous, which is likely related to the high connectivity of the region (7, 9, 10). Insular anatomy is categorized in the antero-posterior and ventro-caudal directions, with different regions responsible for specific semiological signs (23). Patients with insular epilepsy can experience auras, usually with somatosensory sensations or laryngeal constriction. Pain and gustatory sensations also occur. Ictal motor signs are present in most patients (orofacial involvement, dysarthria, hypermotor behaviors, posturing, and eyeblinking) (23, 25, 33). Diagnosis of insular epilepsy requires extensive investigation using techniques such as SEEG, magnet electroencephalography, single photon emission computed tomography and functional MRI (32). The epileptic networks associated with insular epilepsy can be extensive and include additional nodes, typically in the temporal and frontal regions (opercular or central) leading to complex ictal semiology. Due to this patients with insular epilepsy should undergo an extensive work up that includes invasive monitoring, such as SEEG, and possibly combined ablations with multiple laser trajectories, or additional surgical techniques should be considered when the investigations are suggestive of wider onset zones (23).

In our systematic review, 57% of cases treated with LITT for insular epilepsy achieved an Engel class I outcome. Around one third of the patients experienced a temporary paresis. Gireesh et al., reported one intracerebral hematoma following LITT that required a decompressive hemicraniectomy, but subsequently had a good outcome (26, 34). Bouthillier et al., recently published the results of 44 insulo-opercular cortectomies in 43 patients achieving a an Engel class 1 outcome in 77% (mean follow-up 6 years) with a 7% permanent postoperative deficit. While open resections are reported to have a more favorable seizure outcome, 60.9% (*n* = 28) of the LITT cases in our review had previously failed epilepsy surgery which shows how complex the selected cohort may be. Not all patients are eligible for surgery, and LITT may be associated with less permanent side-effects due to the minimally invasive nature of the procedure. In the largest pediatric epilepsy LITT series by Perry et al., 70% of cases were MRI negative and 85% had previously failed surgery (28). Half of the patients in the series achieved an Engel class I outcome with no permanent complications (six mild temporary hemiparesis and one dysphasia). Recently, Hale et al. (25), compared 14 insular LITT cases with 12 open resections and showed similar outcomes. Seizure freedom was achieved in 43% of LITT cases at 1.9 year follow-up with temporary hemiparesis in 46% of cases, while patients who underwent open surgery had seizure freedom in 50% of cases and temporary hemiparesis in 50%. Insular pathologies are not always isolated to the insula and can present with frontal and temporal extensions requiring additional treatment trajectories and sessions. In the series published by Perry et al. (28), seven ablations extended to the opercula. Gireesh et al. (26), reported successful LITT therapy for cingulate and insular epilepsies, including one case with simultaneous LITT in both locations and an Engel class IB outcome. Similar positive experience is apparent from LITT used for temporal lobe epilepsy. Seizure freedom after LITT is achieved in 58% of patients with medically refractory temporal lobe epilepsy, and in 66% of patients with temporomesial sclerosis, compared to 73 and 67% for open anterior temporal lobectomy and selective amygdalohippocampectomy, respectively (35–37).

### Laser interstitial thermal therapy for insular tumors

The surgical resection of insular lesions is a challenge and is associated with a significant functional deficit (39). Two common open surgical options for insular lesions are the trans-sylvian and transcortical approaches with awake brain mapping (40–42). A meta-analysis of eighth studies evaluating 227 patients with insular gliomas showed that permanent neurological deficit was lower in cases with awake mapping (3.5 vs. 15.7%, *p* = 0.001). The rate of early adverse effects was higher in patients operated under general anesthesia (47.7 vs. 27.3%, *p* = 0.04). Laser interstitial thermal therapy offers a minimally invasive alternative therapy for insular tumors. We identified seven patients with insular tumors treated with LITT. Three of the cases had transient aphasia after treatment. Kamath et al. reported 58 glioblastoma treatments with LITT (18 of which were deep in location and two were insular). The average tumor volume was 12.5 ± 13.4 cm^3^. Median overall survival after LITT was 11.5 months for all tumor locations (3). This is comparable to the mean treated insular tumor volume found in our systematic review (12.3 cm^3^). In select cases, laser interstitial thermal therapy may offer a good balance between cytoreduction, and morbidity compared to open resections, but it requires further research with respect to overall survival and occurrence of adverse effects.

### Treatment parameters and trajectories

Hawasli et al., reported treatment of 17 targets using LITT including three insular lesions (two tumors and one epilepsy focus). The mean target volume for all lesions was 11.6 cm^3^. Laser interstitial thermal therapy produced 93% target ablation. In their series, insular lesions required 2 trajectories, the operations lasted 7–8 h, and target volume ranged from 5.8 to 14.6 cm^3^. Patients with deep seated lesions treated with LITT had more complications and a longer ICU stay than superficial ones (22). In the large pediatric series by Perry et al., mean hospitalization time after surgery was 1.8 days (range 1–10). In 63% of the cases, patients were discharged within 24 h and in 88% within 48 h. In 16 cases, LITT ablation required only one trajectory, while four cases required two. Additionally, in seven patients out of 20 it was possible to use the same trajectory directly after the recordings as SEEG. Post-procedure pain was reported to be minimal (28). In our review, ablation parameters and effective doses for insular LITT varied slightly (6–10.5 W for 90–180 s producing target temperatures close to 90°C). Lesion extent was assessed intraoperatively using MR thermography. The mean ablation volume that was achieved was 11.8 cm^3^ for all reported lesions. Lesions with a diameters more than 3 cm might require more than one trajectory for full treatment and large volumes are associated with a higher risk of complications (22). Alexander et al., reported that 1205.86 J of energy to the insula was required to achieve 1 cm^3^ of ablation (27). Insular LITT is technically possible and produces sufficient temperatures both in oncologic lesions and epileptic focus to induce apoptosis and necrosis (22). Similar to MR-guided focused ultrasound procedures, LITT could be performed awake with neurological monitoring for increased safety or as a multistage procedure (31).

Several LITT trajectories are available: the orthogonal approach (OA) with the transopercular placement of axially oriented electrodes, and the oblique approach (ObA). While being shorter, the OA approach is potentially more dangerous due to nearby Sylvian vessels. The parasagittal anterior and posterior ObA approaches are potentially safer, but less accurate due to distance to target and bone drilling angle. Another advantage of the ObA approach is higher coverage of the insular surface. The entry point will depend on the chosen trajectory (ObA vs. OA) and should avoid traversing vessels on the surface of the brain, and in the depth of the sulci. While using the oblique trajectory care should be taken to avoid bridging veins. In the coronal plane, the probe is oriented sufficiently close to the surface of the insula to avoid important subcortical white matter tracts. In case of the orthogonal approach a window is chosen between the veins and MCA branches on the insular surface. The entry point should avoid eloquent cortical regions, such as the frontal operculum and rolandic cortex, and allow sufficient coverage of the target by the laser probes. The trajectory and entry point are individually adjusted according to the patients' insular orientation and vascular anatomy. Similar to SEEG, with wider use of robotics (Neuromate, ROSA), the oblique approaches to the insula are becoming more common (43). The full ablation of the insula generally requires three laser probes and is limited by the spatial organization of the insular gyri and sulci (27).

The insula is limited by the anterior, superior, and inferior peri-insular sulci. Morphologically, the insula is divided into two parts by the central insular sulcus: the anterior part consists of 3–5 short gyri, and the posterior portion consisting of 2–3 long gyri. The insula is encompassed and supplied by the M2 segment and associated branches of the middle cerebral artery, and its course should be always considered while planning trajectories (40). Deep to the surface of the insula are white matter tracts associated with movement, language, and cognition (corticospinal tract, fronto-occipital fascicle and superior longitudinal fascicle) (41). The efficient spread of thermal energy in the middle insula is limited by the central sulcus potentially leaving a remnant that might require additional treatment (44). In cases where the lesions are not affecting the full extent of the insula only one two probes might be sufficient. Using only the anterior and posterior probes oriented according to the long and short gyri creates a sufficient ablation that is bordered by the circular sulcus (Figure 3). Additional experiences and understanding of insular connectivity are needed to guide the safe application of LITT for insular lesions.

## Limitations

The generalization of this systematic review is limited by the non-randomized and observational nature of the studies included in the analysis. Most of the studies included were small case series with two larger pediatric insular epilepsy cohorts published (25, 28). The number of reported tumor treatments remains small. A positive publication bias might occur. Patient cohorts with insular epilepsy treated by LITT are heterogeneous and consist of patients with drug resistant epilepsy, who already often have undergone multiple failed procedures. In cases with no significant improvement after surgery, the possibility of a difficult to localize epileptogenic network remains, highlighting the need for further investigation.

## Conclusion

There is growing evidence supporting the use of LITT in insular epilepsy and tumors, however it remains a novel procedure requiring further studies. Laser interstitial thermal therapy appears to be a safe and a viable surgical option for the treatment of intracranial lesions and may be considered for select patients. Laser interstitial thermal therapy requires shorter hospitalization times than open surgery and therefore could be associated with a decrease in healthcare cost. Laser interstitial thermal therapy, although focal, produces a cytoreductive/ablative measure that can coagulate most of the tumor or epileptic lesion volume in select patients. However, additional studies are necessary to completely evaluate LITT for clinical efficacy and cost effectiveness.

## Data availability statement

The original contributions presented in the study are included in the article/Supplementary material, further inquiries can be directed to the corresponding author/s.

## Author contributions

SK, AV, and JG have conceived the manuscript idea. AV and KY have performed the literature search and extracted the articles. AV has collected the data and drafted the manuscript. JG, AB, NS, CS, BS, CC, CRC, SL, AL, GI, TV, and PK have contributed to the figures, analysis, and manuscript preparation. All authors contributed to the article and approved the submitted version.
